# The enigmatic role of the C-reactive protein and albumin in Behcet’s disease

**DOI:** 10.55730/1300-0144.6172

**Published:** 2025-07-29

**Authors:** Zohreh JADALI

**Affiliations:** Department of Immunology, School of Public Health, Tehran University of Medical Sciences, Tehran, Iran

**Dear Editor**,

I read the article by Pala et al. [1] about the association between C-reactive protein (CRP) to albumin ratio with disease activity in Behcet’s disease (BD) patients. Their findings are important because inflammatory markers are valuable for assessing inflammatory status and predicting disease prognosis. I would like to add a few points that may be clinically relevant.

CRP has two isoforms termed pentameric CRP (pCRP) and monomeric CRP (mCRP).pCRP exhibits antiinflammatory activity, while mCRP shows proinflammatory effects. Research indicates that mCRP is a better marker than pCRP for more accurate diagnosis of inflammatory conditions [2]. There is growing evidence that mCRP can directly participate in and amplifies inflammatory processes through interaction with endothelial cells, leukocytes, and platelets. It also provides a more precise assessment of disease progression compared to pCRP. mCRP accumulation in inflamed tissue makes it a valuable biomarker for measuring the extent of inflammation in various disease states [3]. Therefore, differentiation between CRP isoforms is essential for comprehending the underlying mechanisms of disease.

Albumin is another chief molecule during inflammatory responses which was measured in Pala et al.’s study [1]. It is a negative acute phase reactant, and it has a longer half-life and lower variability than CRP [4]. Therefore, serum albumin (S-Alb) levels could be a good biomarker for inflammatory disorders, and its role has been extensively studied in a wide range of inflammatory diseases including BD. Nonetheless, there are some basic points about the interpretation of albumin results. One of the most important is the concept of ‘effective albumin concentration’ which could have had an impact on interpreting research findings and drawing conclusions. This term refers to the concentration of functionally and structurally intact albumin. Alterations of albumin’s structural and functional features can influence its physicochemical properties and have been reported in a variety of diseases [5]. The underlying mechanisms by which different forms of albumin affect disease are complex. Its native form has been involved in multiple antiinflammatory processes. However, posttranslational modifications of albumin, including oxidation and nitrosylation in an inflammatory milieu, can impair its antiinflammatory activities. Available evidence supports the importance of structural alterations of human S-Alb in patients with BD. Laboratory assessments indicate that the relative abundance of native albumin is significantly reduced in patients with BD [6]. In contrast, these patients exhibit an increase in the levels of oxidative stress markers, including ischemia-modified albumin. Some studies have also shown that these products can increase the risk of developing BD [7]. Therefore, simultaneous determination of the native and modified albumin improves understanding of the role of these molecules in disease development.

Fluctuations in albumin and CRP is another serious issue that must be considered. These molecules are moving targets and can be altered at various stages of the inflammatory responses [8]. For this reason, the lack of repeated sampling of albumin and CRP limit the interpretation of the study results.

Overall, accumulating evidence suggests the role of inflammatory markers, particularly CRP and albumin, in disease progression of BD patients. However, further experimental investigations are required to elucidate the mechanisms underlying this association.
